# Idiopathic generalized epilepsy with phantom absences, absence status, and generalized tonic-clonic seizures: A case report

**DOI:** 10.1097/MD.0000000000035601

**Published:** 2023-11-10

**Authors:** Yongning Jiang, Xiangqin Zhou

**Affiliations:** a Department of Neurology, Dandong Central Hospital, Liaoning, China; b Department of Neurology, Peking Union Medical College Hospital, Beijing, China.

**Keywords:** absence status, case report, generalized tonic-clonic seizure, idiopathic generalized epilepsy, phantom absence

## Abstract

**Rationale:**

Phantom absences refer to mild and short-lasting absence seizures, which are usually accompanied by infrequent generalized tonic-clonic seizures and absence status. Generally, phantom absences do not impair the individual neurological functions. Herein, we report the case of a young woman with idiopathic generalized epilepsy, phantom absences, absence status, and generalized tonic-clonic seizures.

**Patient concerns:**

A 31-year-old woman presented with a 16-year history of paroxysmal convulsions.

**Diagnoses:**

Electroencephalogram (EEG) showed recurrent universal and synchronized 3~4 Hz spike waves and spike-slow waves in the interictal phase with normal background activity. During the ictal phases, EEG revealed bursts of 3~4 Hz spike waves and spike-slow waves that were universal, synchronized, and symmetrical. Additionally, there was 1 seizure episode induced by a 3-Hz flash in the current case. Based on these findings, a diagnosis of idiopathic generalized epilepsy was made.

**Interventions:**

The patient was treated with oral sodium valproate, and the epileptic seizures were controlled.

**Outcomes:**

The frequency of absence seizures was significantly reduced and there were no generalized tonic-clonic seizures.

**Lessons:**

Idiopathic generalized epilepsy with phantom absences, absence status, and generalized tonic-clonic seizures is an extremely rare condition. EEG is the exclusive method for diagnosis. Antiepileptic drugs are effective for controlling epileptic seizures in this disease.

## 1. Introduction

Phantom absences refer to mild and short-lasting absence seizures that are barely noticed by the patient or observers.^[1]^ This disorder was originally described by Panayiotopoulos et al in 1993.^[2]^ Phantom absences are not an isolated seizure form and are usually accompanied by infrequent generalized tonic-clonic seizures and absence status, which can occur in up to 50% of patients).^[3]^ In 1997, Panayiotopoulos et al reported a distinct condition characterized by generalized tonic-clonic seizures, phantom absences, and frequent absence status; the authors considered this peculiar disorder a special subtype of idiopathic generalized epilepsy.^[4]^ The duration of phantom absences is approximately 2~4 seconds, and there are no automatisms or vegetative manifestations.^[4]^ Generally, phantom absences do not impair the individual neurological functions and may lead to transient lapses in concentration and attention.^[4]^ To date, reports of this condition in the literature are extremely rare.^[1,5–8]^ Herein, we report the case of a young woman with idiopathic generalized epilepsy, phantom absences, absence status, and generalized tonic-clonic seizures.

## 2. Case report

**Figure 1.**
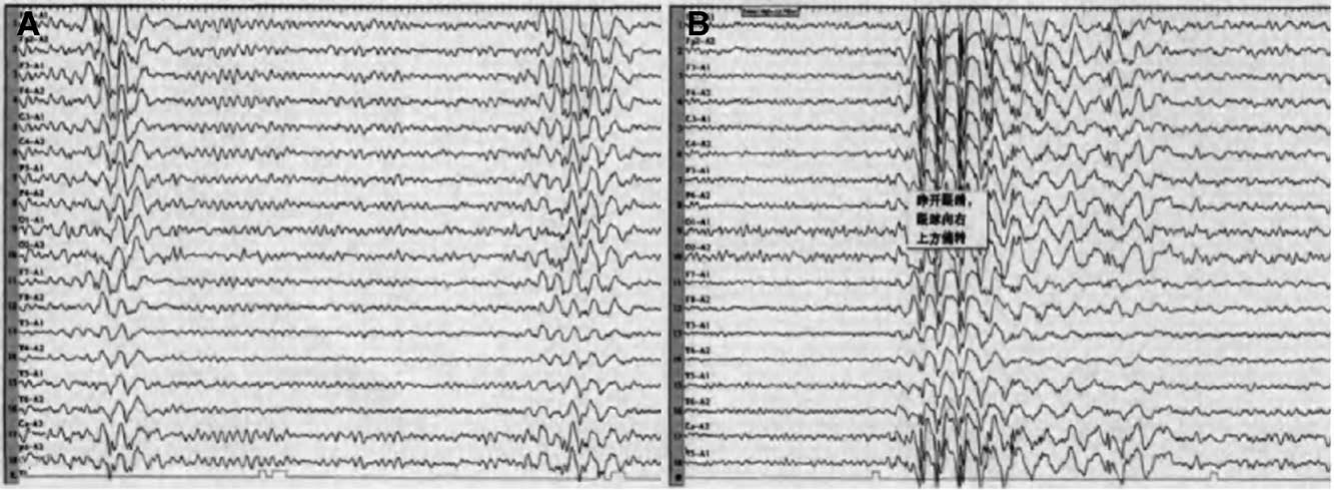
Electroencephalogram. (A) Electroencephalogram showed recurrent universal and synchronized 3~4 Hz spike waves and spike-slow waves in the interictal phase with normal background activity. (B) During the ictal phases, the electroencephalogram revealed bursts of 3~4 Hz spike waves and spike-slow waves.

**Figure 2.**
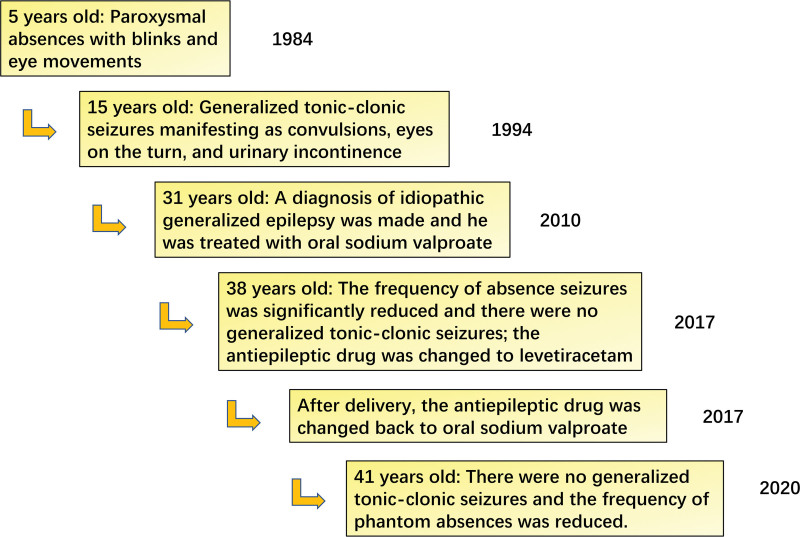
Timeline of the patient stay.

A 31-year-old woman presented to us in 2010 with a 16-year history of paroxysmal convulsions. When the patient was 5 years old, she developed paroxysmal absences with blinks and eye movements. Each episode lasted for approximately 2~4 seconds and there were several to dozens of episodes every day. Her life and studies were not significantly affected. The patient reported experiencing a sudden stop of movement and transient loss of consciousness, but no myoclonic movement. When the patient was 15 years old, she developed generalized tonic-clonic seizures that manifested as convulsions, eyes on the turn, and urinary incontinence. Each seizure lasted for about 1~2 minutes and there were several seizures annually. After the episodes, the patient felt drowsy. There was no history of febrile convulsions or other neurological diseases. The patient denied any family history. Physical and neurological examinations were all normal. Head computed tomography and brain magnetic resonance imaging showed no abnormalities. Electroencephalogram (EEG) showed recurrent universal and synchronized 3~4 Hz spike waves and spike-slow waves in the interictal phase with normal background activity (Fig. 1A). During the ictal phases, EEG revealed bursts of 3~4 Hz spike waves and spike-slow waves that were universal, synchronized, and symmetrical (Fig. 1B); clinical manifestations included sudden eye opening, eye movement upward to the right, and blinks. During the episode, the patient did not respond to voice commands. Each episode lasted for about 4 seconds and she had 1 seizure episode induced by a 3-Hz flash. A diagnosis of idiopathic generalized epilepsy was made and the patient was treated with oral sodium valproate (500 mg twice daily). During the following 7 years, the frequency of absence seizures was significantly reduced and there were no generalized tonic-clonic seizures. In 2017, the patient became pregnant and the antiepileptic drug was changed to levetiracetam (1250 mg twice daily); subsequently, she experienced 2 episodes of generalized tonic-clonic seizures with frequent phantom absences. After delivery, the antiepileptic drug was changed back to oral sodium valproate (500 mg twice daily). There were no generalized tonic-clonic seizures and the frequency of phantom absences was reduced during the following 3 years. In the last year, no seizures were observed. Timeline of the patient stay was presented in Figure 2.

## 3. Discussion

In the current case, all the epileptic seizures were generalized and EEG showed universal spike and spike-slow waves with normal background activity. Additionally, the patient had normal intelligence, and neurological examinations and brain magnetic resonance imaging were all normal. These features supported the diagnosis of idiopathic generalized epilepsy.^[9]^ Absence seizures are mild and do not affect the patient daily life. Panayiotopoulos *et al* proposed the term “phantom absence” for this condition, as the mild and temporary impairment of cognitive function fails to attract the attention of patients, their families, and clinicians.^[4]^ Adult phantom absences do not represent childhood or adolescent absence seizures, which are associated with age or medically treated abortion, because the clinical manifestations of phantom absences are invariable. Phantom absences are usually overlooked prior to the diagnosis.^[5]^

The clinical symptoms of phantom absences are so mild and transient that the affected patients and their families are unaware of the condition and the epileptic seizures. The obvious clinical symptoms that cause a patient to seek medical attention are often generalized tonic-clonic seizures in later adolescence or adulthood. Video EEG monitoring can detect phantom absences when the patient suddenly stops counting or misses counting during hyperventilation. After careful inspection of the medical history, the patient usually admits to experiencing transient attention deficits, thinking hysteresis, or motor arrest. Neuropsychological examination suggests that patients have only mild attention and executive dysfunctions, with impaired selective initial response and voluntary behaviors, while the short-term retention of external information remains intact.^[10]^ Phantom absences do not affect the individual life and studies, and thus are often considered to have no pathological significance.^[4]^ In the current case, the patient relatives had noticed the phantom absences in the early stage, but no medical attention was sought.

The incidence of absence status is 50% in patients with phantom absences.^[5]^ Absence status may appear alone or end with generalized tonic-clonic seizures and the entire course lasts for several hours. During these episodes, patients communicate slowly and exhibit defects in recognition, confusion, and depression, but they can respond to voice commands and recall the course of the seizures. Patients with recurrent absence status may predict the occurrence of generalized tonic-clonic seizures, and they usually try to find a quiet place to lie down and wait for the attack. Pediatric patients presenting with absence status as the onset symptom are rare.^[7]^

EEG is the only method for the diagnosis of phantom absences. An effective, simple, practical, and sensitive strategy for clinical diagnosis is the induction of clinical seizures. During EEG video monitoring, the patients are asked to take hyperventilating breaths and count the number of breaths at the end of exhalation.^[10]^ On EEG, phantom absences manifest as universal and synchronized 3~4 Hz spike waves and spike-slow waves. Additionally, there was 1 seizure episode induced by a 3-Hz flash in the current case, which has never been reported in the literature.

In adults with generalized tonic-clonic seizures and focal spike waves and spike-slow waves on EEG, a diagnosis of focal epilepsy is usually suspected. The disturbance of consciousness prior to generalized tonic-clonic seizures is often considered a symptom of temporal lobe epilepsy. The mild cognitive disorder in absence status may be misdiagnosed as a depressive state. The treatment for idiopathic generalized epilepsy with phantom absences, absence status, and generalized tonic-clonic seizures remains controversial. In previous reports, none of the patients received specific treatments before the occurrence of generalized tonic-clonic seizures. To control generalized tonic-clonic seizures or remarkable absence status, antiepileptic drugs (such as sodium valproate, lamotrigine, and levethrazine) can be adopted. Similar to childhood and adolescent absence seizures, phantom absences respond well to valproate, and the incidence decreases with age. Phantom absences without generalized tonic-clonic seizures should be distinguished from Tourette syndrome in childhood.

In conclusion, idiopathic generalized epilepsy with phantom absences, absence status, and generalized tonic-clonic seizures is an extremely rare condition. EEG is the exclusive method for diagnosis. Antiepileptic drugs are effective for controlling epileptic seizures in this disease.

## Acknowledgments

We would like to thank the patients and their families for their participation in this study. We are equally grateful to the nursing and technical staff members of Peking Union Medical College for their hard work and dedication.

## Author contributions

**Conceptualization:** Yongning Jiang, Xiangqin Zhou.

**Data curation:** Yongning Jiang, Xiangqin Zhou.

**Formal analysis:** Yongning Jiang.

**Funding acquisition:** Yongning Jiang.

**Resources:** Xiangqin Zhou.

**Software:** Yongning Jiang.

**Validation:** Xiangqin Zhou.
